# C5 inhibition with eculizumab prevents thrombotic microangiopathy in a case series of pig-to-human kidney xenotransplantation

**DOI:** 10.1172/JCI175996

**Published:** 2024-01-25

**Authors:** Maggie E. Jones-Carr, Huma Fatima, Vineeta Kumar, Douglas J. Anderson, Julie Houp, Jackson C. Perry, Gavin A. Baker, Leigh McManus, Andrew J. Shunk, Paige M. Porrett, Jayme E. Locke

**Affiliations:** 1 Department of Surgery, Division of Transplantation,; 2 Department of Pathology, and; 3 Department of Medicine, Division of Nephrology, Heersink School of Medicine, University of Alabama at Birmingham, Birmingham, Alabama, USA.; 4 Legacy of Hope, Birmingham, Alabama, USA.

**Keywords:** Nephrology, Transplantation, Complement, Innate immunity, Organ transplantation

**To the Editor:** Although studies of porcine kidney xenotransplantation in nonhuman primates (NHPs) and brain-dead humans have improved our understanding of anti-xenograft immune responses (1–3), the optimal immunosuppression regimen for living human recipients is unknown (3). Prior NHP studies suggest that complement plays an important role in immune-mediated injury of xenografts (4), but the benefits of pharmacologic complement inhibition in human xenograft recipients have yet to be established. Here, we report the histologic outcomes of a series of brain-dead human recipients of a porcine kidney xenotransplant using exclusively FDA-approved medications, with and without anti-C5 monoclonal antibody.

Three male decedents, aged 57, 65, and 53 years, respectively, underwent bilateral native nephrectomies, followed by crossmatch-compatible xenotransplantation (Supplemental Figure 1; supplemental material available online with this article; https://doi.org/10.1172/JCI175996DS1) with 10 gene-edited (10GE) pig kidneys that expressed 2 human transgenes responsible for classical complement cascade inhibition (CD46 and DAF) (2) with standard immunosuppression (Supplemental Methods). There was no evidence of hyperacute rejection in any decedent. Decedents 2 and 3 received anti-C5 monoclonal antibody therapy (eculizumab) 24 hours prior to (1,200 mg) and 24 hours after (900 mg) xenotransplantation.

Decedent 1 native kidney and 10GE porcine donor kidney biopsies were histologically normal without membrane attack complex (MAC; c5b-9) deposition on IHC at the time of transplant (Figure 1 and Supplemental Table 1). On postoperative day 1 (POD1), the xenografts demonstrated evidence of early thrombotic microangiopathy (TMA) along with rare MAC reactivity along capillary loops. By POD3, TMA and MAC deposition were diffuse (Figure 1 and Supplemental Table 1). The observed TMA was seen in the absence of therapeutic tacrolimus levels (POD1&2 <2.0 ng/mL and POD3 3.2 ng/mL; Supplemental Figure 2).

Decedent 2 native kidney biopsies (Figure 1 and Supplemental Table 1) demonstrated no histologic evidence of TMA; however, MAC staining was seen along capillary loops, indicative of complement activation before xenotransplant. Xenograft biopsies prior to implantation and on POD1 and POD3 demonstrated no histologic evidence of TMA or MAC reactivity (Figure 1 and Supplemental Table 1). Tacrolimus remained subtherapeutic (POD1 <2.0 ng/mL; POD2 4.5 ng/mL).

Decedent 3 native kidney showed tubular atrophy and severe arteriosclerosis, consistent with the decedent’s known chronic kidney disease, without evidence of TMA, though MAC deposition was present (Figure 1 and Supplemental Table 1). Xenograft biopsies on POD0, POD1, and POD3 had no MAC deposition, though MAC deposition was observed on POD5 and POD7 (Figure 1 and Supplemental Table 1) in the setting of subtherapeutic eculizumab (Supplemental Figure 3 and Supplemental Table 1). TMA was not observed during the 7-day study period. Tacrolimus was 12 ng/mL on POD1, then ranged from 8.5 to 11.3 ng/mL, before peaking at 19.7 ng/mL on POD7 (Supplemental Figure 2).

We note that tacrolimus levels remained subtherapeutic in Decedents 1 and 2, but Decedent 3 had tacrolimus levels within therapeutic range for much of the study without evidence of TMA and in the presence of normal organ function (5). Altogether, these results suggested that the observed TMA was not the result of exposure to the calcineurin inhibitor, further reinforcing the use of tacrolimus as a maintenance immunosuppressant in the short-term. Importantly, the immunosuppression regimen utilized in Decedents 2 and 3 followed an FDA-approved, standard-of-care regimen that involves complement inhibition at C5 for kidney allotransplant recipients with atypical hemolytic uremic syndrome (aHUS). Similar to human allograft recipients with aHUS, eculizumab levels will likely require surveillance, as indicated in Decedent 3 where subtherapeutic levels of eculizumab correlated with resurgence of MAC deposition (Figure 1 and Supplemental Figure 3).

While complement activation in the setting of both brain death and xenotransplantation is difficult to decipher (3, 6), C5 inhibition may be beneficial in preventing TMA in pig-to-human xenotransplantation. Complement activation in the setting of brain death is common (6) and was observed in Decedents 2 and 3, with the native kidneys staining positively for MAC. However, native kidneys from Decedent 1 had no evidence of complement activation, yet, after xenotransplantation, MAC deposition and TMA progressed rapidly, suggesting an immune response to the xenograft rather than brain death physiology. Prior work by our group has demonstrated development of TMA in the absence of IgM and IgG or C1, C3c, or C4d deposition (2), suggesting activation of the alternative complement cascade via the innate immune system. Given that both the classical and alternative complement cascades converge at C3, downstream complement inhibition at C5 may be necessary to control the innate human immune response to porcine xenografts. Optimal inhibition of the complement cascade in crossmatch-compatible xenotransplant recipients may require inhibition of the alternative complement cascade, especially given the observation of TMA in the absence of therapeutic levels of eculizumab, despite expression of transgenes that inhibit the classical complement cascade (CD46 and DAF) in 10GE xenografts.

In summary, our case series supports utilization of complement inhibition at C5 to control the innate human immune response to porcine kidney xenografts. Because our study reports 3 cases with short-term follow-up, generalizability is limited; however, our findings suggest a beneficial role of anti-C5 monoclonal antibody in pig-to-human kidney xenotransplantation, as previously suggested in the pig-to-NHP model (4). Our case series further demonstrates the utility of the Parsons model in understanding the human immune response to xenografts as well as the short-term efficacy of an FDA-approved, standard-of-care immunosuppression regimen in the setting of 10GE pig-to-human kidney xenotransplantation. Additional studies will be needed to define the long-term utility of this regimen.

## Supplementary Material

Supplemental data

Supporting data values

## Figures and Tables

**Figure 1:**
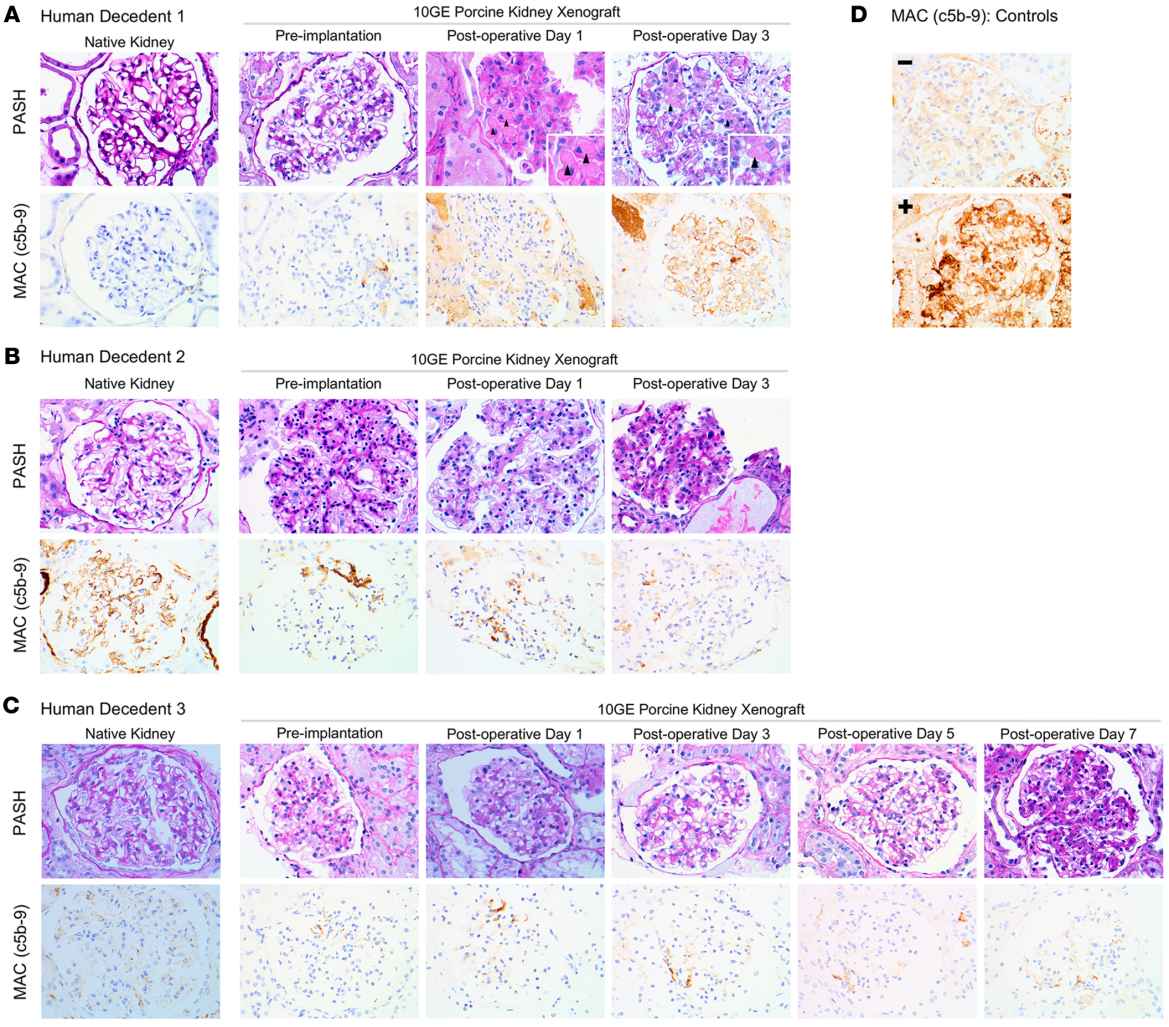
Renal histology and MAC IHC after xenotransplantation of 10GE porcine kidneys using the Parsons model of human decedents. (**A**) Decedent 1; no eculizumab was given. Fibrin thrombi (arrowheads), indicative of TMA. The inset images are roughly original magnification, 60×. (**B** and **C**) Decedents 2 and 3; induction eculizumab was given. Both decedents’ native kidneys were MAC^+^, as well as Decedent 3 xenografts on POD5 and POD7. (**D**) MAC controls (glomerulus specific). All original images: original magnification, ×40.

## Version 1 01/25/2024

In-Press Preview

## Version 2 03/01/2024

Electronic publication

## References

[1] Yamamoto T (2020). Old World Monkeys are less than ideal transplantation models for testing pig organs lacking 3 carbohydrate antigens (Triple-Knockout). Sci Rep.

[2] Porrett PM (2022). First clinical-grade porcine kidney xenotransplant using a human decedent model. Am J Transplant.

[3] Tector AJ (2023). Current status of renal xenotransplantation and next steps. Kidney360.

[4] Adams AB (2021). Anti-C5 antibody tesidolumab reduces early antibody-mediated rejection and prolongs survival in renal xenotransplantation. Ann Surg.

[5] Locke JE (2023). Normal graft function after pig-to-human kidney xenotransplant. JAMA Surg.

[6] Poppelaars F, Seelen MA (2017). Complement-mediated inflammation and injury in brain dead organ donors. Mol Immunol.

