# Supplementary material for: C5 inhibition with eculizumab prevents thrombotic microangiopathy in a case series of pig-to-human kidney xenotransplantation
Source: J Clin Invest. 2024 Jan 25;134(5):e175996. doi: 10.1172/JCI175996 (PMC10904036; doi:10.1172/JCI175996)
Supplement: Supplemental data [file jci-134-175996-s252.pdf]

C5 inhibition with eculizumab prevents thrombotic microangiopathy in a case series of pig-to-human kidney xenotransplantation

Supplementary Material

Table of Contents

Supplemental Methods ……. Page 2

Supplemental Table and Figures …………………………..………………………..……………………………………. Page 3

Acknowledgements …………………..………………………………………………………………………………………….. Page 6

Supplemental Methods

Sex as a biologic variable

Sex as a biologic variable was considered in our study design and as such a specific biologic sex was not excluded from enrollment. However, our study represents a case series, and as such, by random chance no decedents were female.

Data availability

De-identified data used to create the figures have been supplied to the journal as a spreadsheet file, which will be made publically available at the time of publication as the Supporting Data Values file. Additional data are available upon reasonable request to the corresponding author.

Decedent enrollment and porcine donors

The study was approved by the University of Alabama at Birmingham Institutional Review Board for Human Use (No. 300004648) and the Institutional Animal Care and Use Committee (No. 22015). Brain-dead adult decedents precluded from organ donation whose families provided written informed consent for participation were considered for enrollment. Decedents were maintained within a critical care setting on a ventilator. Porcine donors had 10 genetic edits (10GE) (four knockouts: *GTKO, CMAH, B4GALNT2, GHR*; six human transgene insertions: *CD46, CD55* (hDAF, decay accelerating factor)*, CD47, THBD, PROCR, HMOX1*; as previously described) (references 2,5 in manuscript). Two of the transgenes, *CD46* and *CD55*, are human complement inhibitor genes which were inserted to mitigate the effect of complement on the xenografts, primarily via the classical pathway (reference 2 in manuscript).

Immunosuppression

Immunosuppression included induction therapy with methylprednisolone, anti-thymocyte globulin (6 mg/kg total), and anti-CD20 (rituximab). Anti-thymocyte globulin (rabbit) was given in four separate doses (1.5 mg/kg), the first in the operating room and subsequent doses on post-operative days 1, 2, and 3. Rituximab was dosed at 375 mg/kg/m^2 and given 12 hours before xenotransplantation. Maintenance therapy included tacrolimus, mycophenolate mofetil, and prednisone.

Biopsy protocol

Native kidneys were sent for pathologic evaluation, and xenograft biopsies were performed pre-implantation and every other day until termination. Specimens were fixed, stained with periodic acid-Schiff hematoxylin (PASH) and immunohistochemistry (IHC) for membrane attack complex (MAC, C5b-9; Arkana Laboratories, Abcam, #ab66768). Pathologic evaluation was performed by a renal pathologist.

Supplemental Figures

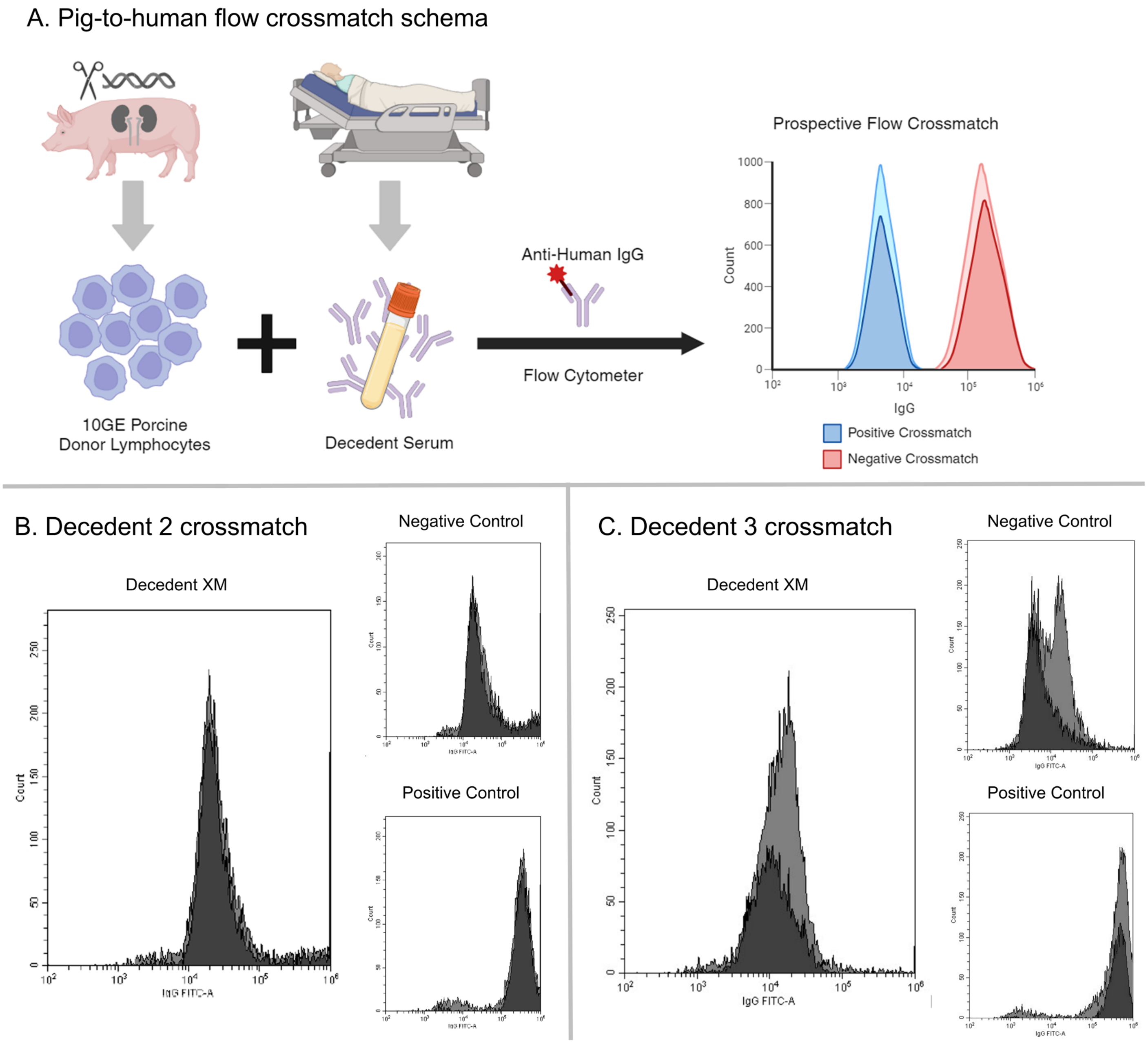

Supplemental Figure 1 (Fig. S1). Crossmatch for pig-to-human xenotransplantation. **A.** Crossmatch schema, as outlined in our prior work (reference 2 in manuscript; Porrett PM, Orandi BJ, Kumar V, et al. First clinical-grade porcine kidney xenotransplant using a human decedent model. *Am J Transplant*. 2022;22(4):1037-1053), *where the Decedent 1 crossmatch can also be found.* Flow cytometry with decedent sera and porcine donor lymphocytes were performed. For all tubes, lymphocytes and serum were incubated with fluorescein isothiocyanate conjugated goat anti-human IgG F(ab)'2; Manufacturer: Jackson ImmunoResearch Laboratories. Pooled sera from human males blood type AB was used as a negative control. Human serum containing IgG known to react with porcine lymphocytes was used as a positive control. **B.** Negative crossmatch for Decedent 2, with appropriate controls. **C.** Negative crossmatch for Decedent 3, with appropriate controls.

Summary of Pathologic Findings

	Native	*Pre-Implantation*	*POD 1*	*POD 3*	*POD 5*	*POD 7*
**Decedent 1**						
Eculizumab		-	-			
Histology	Normal	No TMA	(+)	(++)	-	-
MAC	(-)	(-)	(+)	(++)	-	-
**Decedent 2**						
Eculizumab		Given 24h prior	Given 24h after			
Histology	Normal	No TMA	No TMA	No TMA	-	-
MAC	(+)	(-)	(-)	(-)	-	-
**Decedent 3**						
Eculizumab		Given 24h prior	Given 24h after		Subtherapeutic	Subtherapeutic
Histology	*Abnormal	No TMA	No TMA	No TMA	No TMA	No TMA
MAC	(+)	(-)	(-)	(-)	(+)	(+)

Supplemental Table 1. Summary of pathologic findings in relation to eculizumab dosing. POD = post-operative day, MAC = membrane attack complex, TMA = thrombotic microangiopathy. Eculizumab was dosed 24 hours prior to xenotransplantation and again 24 hours after.

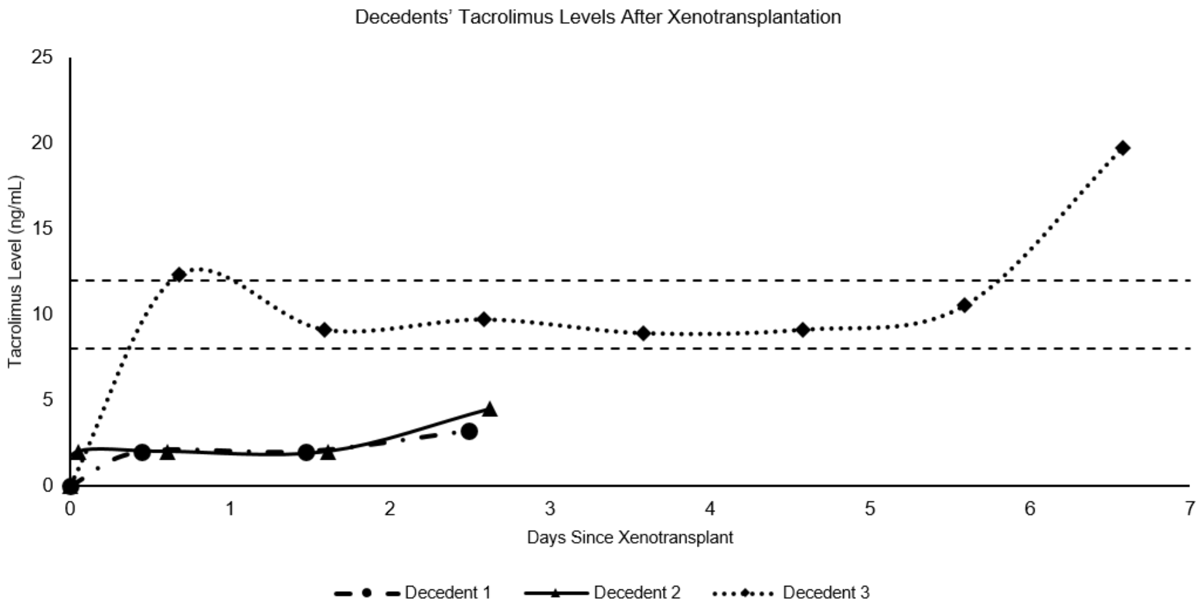

Supplemental Figure 2. Decedents' tacrolimus levels following xenotransplantation. Note only Decedent 3 reached therapeutic range (8-12 ng/mL, dashed lines).

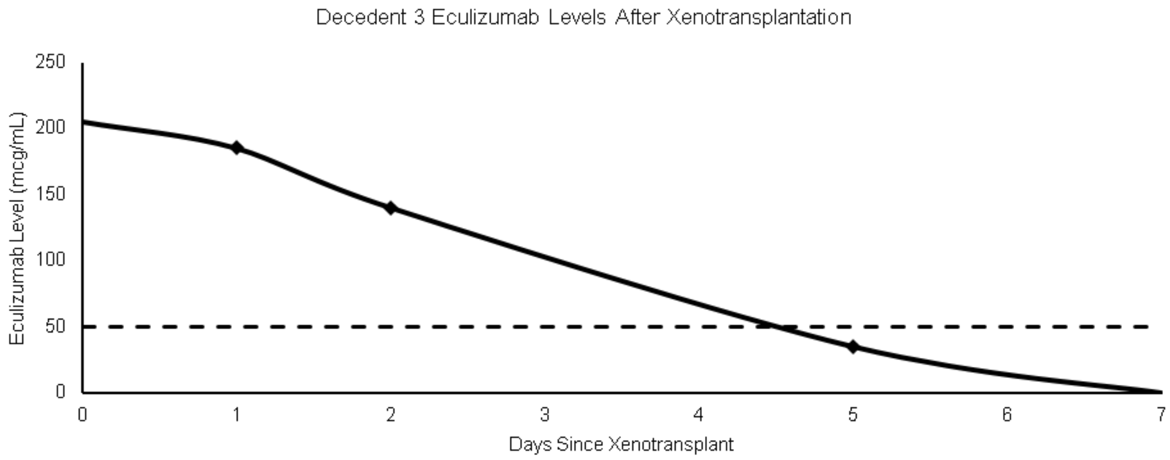

Supplemental Figure 3. Eculizumab level of Decedent 3 after xenotransplantation. Note the subtherapeutic levels at post-operative day 5, when MAC staining became evident (Figure). >50 mcg/mL is the published therapeutic threshold for atypical hemolytic uremic syndrome (aHUS, dashed line), Mayo Clinic Laboratories.

Acknowledgements:

We would like to acknowledge the extraordinary courage of our donor families, the James Parsons Family, James Willis Family, and those who wish to remain anonymous. Their loved ones are true pioneers and will be forever remembered for the lives they have so positively impacted.

We would also like to recognize the contributions of our entire xenotransplant team for making this research possible:

- Pathogen-free facility: Tracy Gamblin; Paul Symms; Deborah Quenell; Daniel Funderburg; Jeff Hendrix; Linda Guy
- UAB Medicine Transplant OR Team: Katie Stegner; Natalie Budd; Sara Macedon; Sandi Jo Minor; Loren Brook Eichelberger; Joe Pate; Christian Nichols; Chris McClure
- UAB Heersink School of Medicine Xenotransplant Research Team: Eric Judd; Kelly Hyndman; Vera Hauptfeld; Julie Houp; Matthew Cheung; Rebecca Asiimwe; Shawn Little; Daniel Epstein; Morgan Greene, Stefani Yates, Markayla Bell, Brittney Knott, Dalton Patterson, James F. George, Anupam Agarwal, Cozette Killian, Rhiannon Reed.
- Legacy of Hope (Organ Procurement Organization): Lindsay Banks; Jordan Barksdale; Kellie Bishop; Angela Brown; Kelbi Carman; Rhiannon Carter: Jennifer Clingingsmith; Josh Davis; Sherri Donahue; Alex Feltman; Angela Haffarnan; Caleb Henry; Amy Johnson; Kera Leverette; Kristy Lowe; Rachel Patrick; Brittany Price; Lawanna Salley; Stefanie Salter; Stacy Sexton; Amanda Smith; Maggie Tarpley; Hollana Wood
- Revivicor: Amy Dandro; Maria Kokkinaki; Kasinath V. Kuravi; David Ayares
    - This funder had no role in study design or conduct; data collection, management, analysis, interpretation; manuscript preparation or review; or in the decision to publish the manuscript.

Other acknowledgements: Supplemental Figure 1A was created with BioRender.com.
